# An evaluation of Innowave MTB/RIF/INH assay versus Xpert MTB/RIF for rapid identification of *Mycobacterium tuberculosis* and drug resistance in bone and joint specimens from people with suspected osteoarticular tuberculosis

**DOI:** 10.1128/spectrum.00267-26

**Published:** 2026-05-18

**Authors:** Qiang Liu, Fen Wang, Haoran Wang, Guanglu Jiang, Adong Shen, Hairong Huang, Suting Chen

**Affiliations:** 1 National Clinical Laboratory on Tuberculosis, Beijing Key Laboratory on Drug-resistant Tuberculosis Research, Beijing Chest Hospital, Capital Medical University, Beijing Tuberculosis and Thoracic Tumor Institute 117550, Beijing, China; 2 Kunming Children's Hospital, Kunming Medical University 71240, Kunming, China; End TB Dx Consulting LLC, San Diego, California, USA; Asan Medical Center, Seoul, Republic of Korea; Saban Research Institute of Children's Hospital Los Angeles, Los Angeles, California, USA

**Keywords:** osteoarticular tuberculosis, diagnosis, drug resistance, rifampicin, isoniazid

## Abstract

Osteoarticular tuberculosis (TB) is hard to diagnose. We prospectively evaluated a novel molecular test, Innowave MTB/RIF/INH (InnowaveDX), against culture and Xpert MTB/RIF (Xpert)/MeltPro TB for detecting *Mycobacterium tuberculosis* (MTB) and rifampicin and isoniazid resistance in osteoarticular TB. Pus/tissue samples from suspected osteoarticular TB patients were tested by smear, culture, Xpert, and InnowaveDX; drug-susceptibility testing was conducted on MTB isolates. A comprehensive reference standard (CRS), which included clinical, laboratory, histopathological, and radiological data, was used as the gold standard. From June 2024 to January 2025, 150 suspected osteoarticular TB patients were enrolled. Eighty patients met CRS-TB criteria. The sensitivity of InnowaveDX for MTB detection was 95% (76/80), surpassing Xpert (87.5%), culture (35%), and smear (16.25%). Both InnowaveDX and Xpert correctly identified 4 rifampicin-resistant and 22 sensitive cases. For isoniazid, InnowaveDX detected 6/8 resistant and 20/20 sensitive cases, versus 2/3 and 12/14 for MeltPro TB. The concordance of InnowaveDX with phenotypic DST was 94.7% both for the resistance diagnosis of rifampicin and isoniazid. InnowaveDX detects significantly more osteoarticular TB cases than Xpert or culture and simultaneously identifies rifampicin and isoniazid resistance, enabling rapid diagnosis and tailored therapy.

**IMPORTANCE:**

This study shows that Innowave MTB/RIF/INH is a faster and more sensitive test for diagnosing osteoarticular tuberculosis (TB) than current methods. Compared with culture and Xpert MTB/RIF, Innowave detects more TB cases from bone and joint samples, which usually contain very few bacteria. Importantly, it can simultaneously identify resistance to rifampicin and isoniazid within a few hours, instead of waiting weeks for culture-based results. This allows doctors to start the right anti-TB treatment earlier, reduce unnecessary empirical therapy, and lower the risk of joint destruction and disability. Overall, Innowave offers a practical solution to improve early diagnosis and drug-resistance detection in difficult-to-diagnose extrapulmonary TB.

## INTRODUCTION

Tuberculosis (TB), caused by *Mycobacterium tuberculosis* (MTB), remains the deadliest single-pathogen infection, causing an estimated 1.25 million deaths in 2023 (1). Extrapulmonary tuberculosis (EPTB) represents approximately 16% of the 10.8 million new diagnosed TB cases annually (2). Osteoarticular TB accounts for 10%–15% of extrapulmonary disease, yet its paucibacillary nature delays bacteriological confirmation, allowing irreversible joint destruction and vertebral collapse to occur (3–5). Conventional workflow—smear, weeks of culture, and phenotypic drug-susceptibility testing (DST)—misses 60%–80% of true cases and provides resistance data only after 4–8 weeks (5, 6), forcing clinicians to start empirical therapy and risking amplification of resistance. Thus, continuous advancements in the diagnostic methods for osteoarticular TB are critically important.

Molecular diagnostics for TB have expanded rapidly over the past decade, yet more methods with better performance are still needed. Xpert MTB/RIF (Cepheid, USA)—the first cartridge to combine MTB detection with rifampicin (RIF)-resistance prediction—compressed laboratory turnaround to approximately 2 h by five fluorogenic probes targeting the 81 bp *rpoB* core region. Hailed as a “milestone” tool, it was endorsed by WHO in 2013 as the initial screening test for pulmonary TB and was subsequently extended to EPTB (7). Meta-analyses restricted to osteoarticular pus, tissue, and synovial fluid show pooled sensitivity of 81% (95% CI 77–84) and specificity of 99% (95% CI 97–100), although sensitivity falls sharply when bacterial load drops below 10 colony-forming units (CFU)/mL (8–11). Against the traditional proportion-method DST, Xpert MTB/RIF achieves 94.1%–98.2% sensitivity and 90.8%–97.0% specificity for RIF resistance, with κ ≥ 0.9, making it a reliable surrogate for DST (12–14). RIF resistance is considered as a marker of multidrug resistance (MDR) (15). Nevertheless, 10%–25% of strains that are RIF resistant are still susceptible to isoniazid (INH). Therefore, this prediction strategy could cause mislabeling and consequently expose patients to prolonged, toxic second-line regimens (16–19). Accurate INH determination is therefore essential once RIF resistance is flagged, yet no WHO-endorsed cartridge simultaneously interrogates the susceptibility of both drugs.

More recently, several commercial assays have added INH detection, but each introduces new trade-offs. The MeltPro TB assay (Zeesan Biotech, China) uses post-PCR melting-curve analysis to detect resistance to key first- and some second-line anti-TB drugs. However, its open-platform workflow requires off-board DNA extraction and manual pipetting, and pooled data show sensitivity for MTB diagnosis as low as 57.6% in paucibacillary specimens, limiting its adoption (20). Fully integrated alternatives—Abbott RealTime MTB RIF/INH (Abbott, USA), BD MAX MDR-TB (Becton Dickinson, USA), and cobas MTB-RIF/INH (Roche, Switzerland)—combine automated nucleic acid extraction with multiplex real-time PCR, achieve INH sensitivity of ≥85%, and are WHO-endorsed for sputum, yet none is commercially available in China, and all require high-throughput laboratory infrastructure.

The Innowave MTB/RIF/INH (InnowaveDX) assay (Chuanglan Ltd., Suzhou, China) was therefore designed to bridge this gap: it retains the closed-tube, sample-to-answer architecture familiar to Xpert users while adding multiple-copy IS6110 to increase sensitivity and including multiple fluorogenic probes for *rpoB*, *katG*, *inhA*, and *ahpC* in a single closed tube, delivering simultaneous RIF and INH resistance results within 3 h on either a dedicated cartridge station or an open PCR platform. Moreover, our team’s earlier research has shown that InnowaveDX is an easy, rapid, and sensitive molecular test for pulmonary TB diagnosis (21). The objective of the present study is (i) to compare InnowaveDX and Xpert in terms of diagnostic sensitivity and specificity for MTB detection, and (ii) to assess the additional value of InnowaveDX in simultaneously detecting INH resistance, in osteoarticular TB, a paucibacillary disease where rapid MDR determination is particularly critical.

## MATERIALS AND METHODS

### Research design and participants

Between June 2024 and January 2025, adult patients clinically suspected of having osteoarticular TB were prospectively enrolled at Beijing Chest Hospital. Inclusion criteria comprised local symptoms (joint pain, swelling, tenderness, effusion, and limited mobility) and/or systemic features (fever, weight loss, and elevated erythrocyte sedimentation rate) together with any TB-associated sign (cough, dyspnea, and previous TB). Post-operative pus or tissue from each participant was concurrently examined by smear microscopy, MGIT culture, Xpert MTB/RIF, InnowaveDX, histopathology, and routine biochemistry. Phenotypic DST was performed on all culture-positive isolates.

### Patient categories

Patients were assigned to one of three categories according to a comprehensive reference standard (CRS) that integrated clinical, laboratory, histopathological, radiological, and follow-up data: (i) confirmed osteoarticular TB: bacteriological evidence was obtained through smear microscopy, culture, or Xpert MTB/RIF; (ii) probable/possible osteoarticular TB: bacteriological evidence was negative; clinical symptoms, imaging manifestations, and histopathological examinations suggested it might be TB; or during the follow-up period, the patient responded well to empirical anti-TB treatment; (iii) non-TB: alternative diagnosis established or clinical improvement without anti-TB treatment.

### Smear and culture

Direct smears were prepared, stained with auramine O, and read under fluorescence microscopy.

After mechanical homogenization, pus or tissue was decontaminated with N-acetyl-L-cysteine–NaOH, centrifuged, resuspended in MGIT960 culture medium, and incubated in the MGIT960 system (BD, USA). Positive cultures were confirmed as MTB complex by MPT64 antigen detection.

### Xpert MTB/RIF assay

Following the manufacturer’s protocol, 1 mL of sample was mixed with 2 mL of sample reagent, vortexed for 10 s, incubated at room temperature for 10 min, revortexed for 10 s, and incubated for a further 5 min, and 2 mL of the liquefied material was transferred to the Xpert cartridge and loaded into the GeneXpert instrument.

### InnowaveDX test

The kit employs real-time fluorescent PCR technology for the qualitative detection of nucleic acids from the MTB complex and any mutations conferring resistance to RIF and INH in specimens. To begin, 1 mL of the sample was mixed with 6 mL of lysis solution. This mixture was then placed at the center of the ultrasonic probe for automatic fixation and ultrasonic lysis. Following the manufacturer’s instructions, nucleic acids were extracted and dissolved in 400 µL of elution solution. For the PCR reaction, 25 µL of the extracted nucleic acid elution solution was added to each reaction tube and mixed thoroughly. The PCR reaction was performed once the Innowave PCR reagent mix had completely thawed. The minimum detection limit for Mtb is 10 bacteria/mL. For detecting RIF resistance, the minimum limit is 1,000 CFU/mL, and the same minimum limit of 1,000 CFU/mL applies for INH resistance.

### MeltPro TB test

The MeltPro TB test was carried out in accordance with the manufacturer’s protocol (14). Genomic DNA was extracted from the isolate with an automated paramagnetic-particle extractor (Zeesan Biotech). Subsequent amplification and high-resolution melting were run on a SLAN-99S Real-Time PCR platform (Hongshi Technology, China); peak calling yielded the Tm, and genotype assignment was based on differences between these Tm values.

### Drug-susceptibility testing

The drug susceptibility profile of isolates was tested by the microplate dilution method. Breakpoint concentrations were 0.2 µg/mL for INH and 0.5 µg/mL for RIF.

### Statistical analysis

Statistical analyses were performed with SPSS 21.0 (IBM, USA). Sensitivity, specificity, positive predictive value, and negative predictive value were calculated against the CRS or other defined categories (see Table 3). Continuous variables were compared with the independent-sample *t*-test or the Mann-Whitney *U* test, and categorical variables were compared with the Pearson χ² or the Fisher exact χ² test when >20% of expected counts were <5. A *P* value of <0.05 was considered statistically significant.

## RESULTS

### Patient characteristics

**Fig 1:**
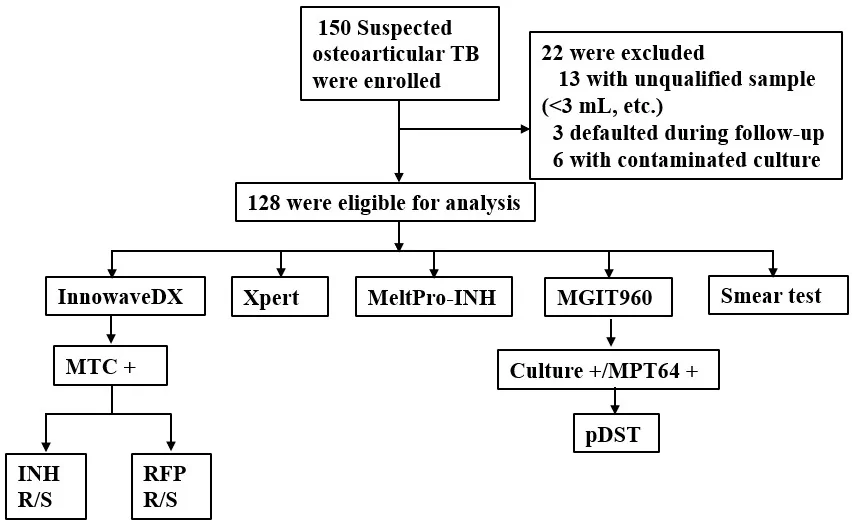
Charts of the diagnosis evaluation of InnowaveDX in suspected osteoarticular TB.

After applying the inclusion and exclusion criteria, 128 adults with suspected osteoarticular TB were included in the analysis (Fig. 1 and Table 1). Eighty subjects (62.5%) fulfilled the CRS for osteoarticular TB: 70 were bacteriologically confirmed, and 10 were classified as probable/possible. The remaining 48 participants (37.5%) received alternative diagnoses: 36 pyogenic infections, 5 rheumatoid arthritis cases, and 7 malignancies, and were classified as non-TB.

**TABLE 1:** Demographic and clinical characteristics of the participants

Characteristics	Osteoarticular TB (*n* = 80)	Non-TB (*n* = 48)	*P* value
Age (year), median (range)	58 (19–86)	63 (37–83)	0.01
Gender, *n* (%)			0.285
Male	47 (58.75)	33 (68.75)	
Female	33 (41.25)	15 (31.25)	
Underlying condition, *n* (%)			
Diabetes mellitus	15 (18.75)	12 (25)	0.401
Hypertension	16 (20)	13 (27.08)	0.354
Rheumatologic disease	4 (5)	2 (4.17)	0.817
Combined TB, *n* (%)			0.016
Pulmonary infection	12 (15)	2 (4.17)	
Pleurisy	4 (5)	0 (0)	
Other	2 (2.5)	1 (2.08)	
Previous TB history			
Yes	5	3	1
Infected site, *n* (%)			0.067
Spine	51 (63.75)	38 (79.17)	
Bone and joint	29 (36.25)	10 (20.83)	
Sample type, *n* (%)			0.100
Tissue	4 (5)	7 (14.58)	
Pus	76 (95)	41 (85.42)	

Concomitant pulmonary TB was observed in 22.5% of the osteoarticular TB group versus 6.25% of the non-TB group; this difference reached statistical significance (*P* = 0.016). No other baseline comorbidity differed between the two groups, and none of the participants were HIV positive. Full demographic and clinical details are presented in Table 1.

### Diagnostic performance of InnowaveDX for osteoarticular TB

Among the 80 CRS-positive osteoarticular TB patients, MTB was detected in 13 by smear test (AFB), 28 by culture, 70 by Xpert MTB/RIF, and 76 by InnowaveDX. InnowaveDX exhibited a sensitivity of 95% (76/80), exceeding that of Xpert MTB/RIF (87.5%, 70/80; *P* = 0.005), culture (35%, 28/80; *P* < 0.001), and smear (16.25%, 13/80; *P* < 0.001) (Table 2). Specificities were 100% (48/48) for culture and Xpert, and 93.75% (45/48) for smear and InnowaveDX (Table 2). There are three false-positive results in InnowaveDX. The final diagnoses for the three cases that were incorrectly identified as positive by InnowaveDX are as follows: one case involved suppurative spondylitis caused by *Propionibacterium acnes* infection; another was a case of suppurative arthritis caused by *Klebsiella pneumoniae* infection; and the third case was a secondary malignant tumor of the sacrum.

**TABLE 2:** Diagnostic accuracies of different methods compared to the composite reference standard^*c*^

Methods	Sensitivity (%)	Specificity (%)	PPV (%)	NPV (%)
Smear	13/80 (16.25)^*b*^	45/48 (93.75)	13/16 (81.25)	45/112 (40.18)^*a*^
Culture	28/80 (35)^*b*^	48/48 (100)	28/28 (100)	48/100 (48)^*a*^
Xpert	70/80 (87.5)^*a*^	48/48 (100)	70/70 (100)	48/58 (82.76)
InnowaveDX	76/80 (95)	45/48 (93.75)	76/79 (96.2)	45/49 (91.84)

^*a*^ Represents statistical significance (*P* < 0.05) when compared with InnowaveDX.

^*b*^ Represents statistical significance (*P* < 0.001) when compared with InnowaveDX.

^*c*^ NPV, negative predictive value; PPV, positive predictive value.

Out of the 70 specimens that tested positive with Xpert MTB/RIF, only one sample was negative in the InnowaveDX assay. The Xpert test indicated low DNA levels for this particular sample (Table 3). Addition of InnowaveDX reclassified 7 of the 10 probable/possible cases (70%) into the confirmed category, raising the proportion of confirmed osteoarticular TB from 87.5% (70/80) to 96.25% (77/80). Overall, 77 osteoarticular TB patients yielded at least one positive result among smear, culture, Xpert MTB/RIF, or InnowaveDX. Among these, seven cases yielded positive outcomes only by InnowaveDX, one by Xpert MTB/RIF, but none by smear or culture.

**TABLE 3:** Diagnostic efficiency of InnowaveDX in different osteoarticular tuberculosis subgroups

Patient groups	Sensitivity (%)			
	Smear	Culture	Xpert	InnowaveDX
Smear+	–^*a*^	7/13 (53.85)	13/13 (100)	13/13 (100)
Culture+	7/28 (25)	–	28/28 (100)	28/28 (100)
Xpert+	13/70 (18.57)	28/70 (40)	–	69/70 (98.57)
High	0/1 (0)	1/1 (100)	–	1/1 (100)
Medium	7/10 (70)	7/10 (70)	–	10/10 (100)
Low	5/43 (11.63)	15/43 (34.88)	–	42/43 (97.67)
Very low	1/16 (6.25)	5/16 (31.25)	–	16/16 (100)
Culture+/Xpert+	7/28 (25)	–	–	28/28 (100)
Culture−/Xpert+	6/42 (14.29)	–	–	41/42 (97.62)

^*a*^ –, not applicable.

In this study, three cases were found to be positive in the smear test; however, the results from both the culture and Xpert tests were negative. Based on the subsequent treatment outcomes of these patients, it was speculated that they may have been infected with non-tuberculous mycobacteria. As a result, these three patients were categorized into the non-tuberculosis case group for analysis. Additionally, the InnowaveDX test for these three samples also returned negative results.

### Performance of InnowaveDX in detecting RIF resistance

Of the 76 patients with osteoarticular TB who tested positive using InnowaveDX, 10 cases were identified as RIF resistant. Additionally, 69 specimens provided interpretable positive nucleic acid results with Xpert MTB/RIF. Both InnowaveDX and Xpert MTB/RIF predicted RIF sensitivity in 59 cases and resistance in 9 cases, giving an overall concordance of 98.55% (68/69). The single discordant result was classified as resistant by InnowaveDX but sensitive by Xpert MTB/RIF; this specimen remained culture negative, precluding phenotypic resolution.

A total of 28 patients had culture-positive results and subsequently underwent phenotypic DST. InnowaveDX and Xpert MTB/RIF each generated valid resistance predictions for every isolate. Against phenotypic DST, both InnowaveDX and Xpert MTB/RIF correctly identified the four RIF-resistant and 22 RIF-sensitive strains, resulting in sensitivity and specificity of 80% (4/5) and 95.65% (22/23) for the detection of RIF resistance.

### Performance of InnowaveDX in detecting INH resistance

For INH resistance detection, InnowaveDX identified 16 cases of isoniazid-resistant and 60 cases of isoniazid-sensitive osteoarticular TB. These 76 patients also underwent MeltPro TB test. The MeltPro TB test is capable of detecting Mtb as well as isoniazid resistance. Therefore, it serves as a comparative method to assess the efficacy of the InnowaveDX in detecting isoniazid resistance. Among the 43 samples that were positive by both assays, 31 were classified as INH susceptible and six as INH resistant, resulting in an overall concordance of 86.05% (37/43). There were six discordant results in total: two isolates were deemed INH resistant by MeltPro TB but INH susceptible by both InnowaveDX and phenotypic DST; the remaining four isolates were scored INH resistant only by InnowaveDX, and these samples were culture negative, preventing phenotypic confirmation.

Phenotypic DST for INH were performed on all 28 positive culture samples, with the InnowaveDX yielding concordant results for each of these samples. According to the phenotypic DST of INH resistance, Innowave demonstrated a sensitivity of 75% (six out of eight) and a specificity of 100% for INH resistance. Additionally, the concordance of Innowave results with the phenotypic DST was 92.86% (26/28). Among the 28 samples, 27 were also tested using Meltpro INH. However, the concordance of the Meltpro INH assay was only 62.96% (17 out of 27). Further analysis of the molecular drug susceptibility results for the positive samples showed that the consistency between the Meltpro INH and the phenotypic DST was 82.35% (14 out of 17).

## DISCUSSION

Osteoarticular TB is a disease that can cause skeletal deformity and irreversible neurological sequelae, leaving patients with permanent cosmetic and functional disability (22, 23). Early bacteriologically confirmed diagnosis and timely therapy are therefore essential to avert these devastating outcomes. InnowaveDX couples real-time fluorescent PCR to multicopy IS6110 and multiprobe *rpoB*/*katG*/*inhA*/*ahpC* interrogation, thereby delivering simultaneous detection of MTB complex and prediction of RIF and INH resistance within a single workflow of 2–3 h (21). The present prospective study is the first to evaluate the incremental value of this integrated cartridge in paucibacillary osteoarticular samples collected under routine clinical conditions.

InnowaveDX achieved a significantly greater sensitivity than Xpert and culture. The superiority persisted across the full spectrum of bacterial load: among the 10 “probable/possible” cases, InnowaveDX reclassified 7 (70%) into the bacteriologically confirmed stratum, raising the proportion of confirmed osteoarticular TB from 87.5% to 96.25%. This observation mirrors the experience in pulmonary specimens, where InnowaveDX maintained 52.73% sensitivity in probable pulmonary TB patients, outperforming both culture and Xpert MTB/RIF (21). The multicopy IS6110 target (1–25 copies/genome) underpins this performance by generating a higher fluorescent signal than single-copy templates when only a few bacilli are present in synovial tissue or necrotic bone (24). Importantly, the assay retained its speed advantage: the median time from sample loading to result was 2–3 h, compared with 4–6 weeks for phenotypic DST. Earlier microbiological confirmation not only permits prompt initiation of appropriate anti-TB therapy but also curtails unnecessary antibiotic exposure and reduces the risk of progressive joint destruction and lifelong disability.

Drug-resistant MTB is gradually emerging as a new challenge in osteoarticular TB treatment. Traditional phenotypic DST is highly dependent on culture; however, osteoarticular TB has a significant paucibacillary characteristic. The positive rate of culture in osteoarticular TB is low (in this study, it was only 35%), and the reporting period is long (4–8 weeks), which leads to an extended diagnostic window. Patients often receive empirical first-line treatment during the waiting period for the results, which not only increases the risk of drug-resistant bacteria transmission but also raises the probability of sequelae, such as bone destruction, deformity, and permanent neurological dysfunction. In the present study, only a total of 28 patients obtained culture positive results and then conducted the phenotypic DST. The traditional phenotypic DST revealed that only five cases of MDR and three cases of INH monoresistance could be identified. However, InnowaveDX identified a total of 76 patients with osteoarticular TB. Among these, there were eight cases with MDR TB, two cases that were resistant to RIF but sensitive to INH, and eight cases of INH monoresistance. InnowaveDX identified RIF resistance with 80% sensitivity and 95.65% specificity and INH resistance with 75% sensitivity and 100% specificity, figures that aligned well with phenotypic DST. This finding corroborates a recent multicenter study of 535 Xpert-positive pulmonary TB cases in which InnowaveDX MTB/RIF reached a sensitivity of 86.4% and a specificity of 94.1% for RIF resistance (25). Subanalysis revealed that 22% of discordant samples carried “very low” bacillary loads by Xpert MTB/RIF, a proportion almost twice that of the concordant subset (9.7%, *P* = 0.029) (25). The combination of high-copy IS6110 signal amplification and ultrasonic lysis thus appears to compensate for DNA insufficiency, a benefit that is especially relevant to osteoarticular specimens where smear-positive cases are seldom encountered. However, these findings should be interpreted with caution due to the limited sample size for drug resistance analysis. The statistical power for detecting resistance, particularly for INH, where the number of resistant isolates was small, was constrained, resulting in wide confidence intervals around sensitivity estimates. Consequently, while these preliminary results suggest that InnowaveDX may have potential predictive value for MDR detection, further validation with larger, adequately powered cohorts is warranted to confirm its diagnostic performance.

The global 2023 WHO TB report estimates that only 46% of RIF-resistant cases are diagnosed and treated appropriately within 4 months of the onset of symptoms (1). In cases of EPTB, this percentage is likely even lower. InnowaveDX addresses a critical gap in the osteoarticular TB care process by reducing the diagnostic interval from weeks to hours and providing results for RIF and INH susceptibility simultaneously. Rapid identification of MDR-TB at the point of biopsy enables clinicians to initiate an optimized treatment regimen before irreversible bone erosion or spinal cord compression occurs.

Our study has several limitations. First, the single-center design and modest sample size limit the generalizability of our findings. Additionally, the low culture yield made it difficult to objectively compare the specificity of INH against a larger phenotypic reference group. Long-term outcome data are essential to determine if earlier resistance-directed therapy leads to significant reductions in deformity and disability. Moreover, there are two commercially available formats of the InnowaveDX assay: a fully automated, cartridge-based version that operates on a dedicated instrument (similar to the Xpert platform) and an open-format version compatible with generic real-time PCR systems, which requires several manual steps. The current assessment focuses on the open-format of InnowaveDX. While its sensitivity is greater than that of Xpert, its specificity is slightly lower (93.5%). This open format of the molecular detection method may present certain limitations, including potential laboratory contamination. These factors could contribute to false-positive results. We anticipate that adopting the automated, closed-tube configuration will help reduce some occasional false-positive signals.

In conclusion, InnowaveDX delivers superior sensitivity for osteoarticular TB detection while furnishing same-day RIF and INH resistance detection. By shortening the diagnostic-to-treatment interval from weeks to hours, the assay offers a pragmatic solution to the “low-bacterial load” dilemma that has long impeded the management of osteoarticular TB. Integration of InnowaveDX into routine orthopedic and spinal surgery workflows is expected to facilitate earlier targeted therapy, curtail empirical broad-spectrum antibiotic use, and ultimately improve musculoskeletal and neurological outcomes for patients with this debilitating form of TB.

## Supplementary Material

Reviewer comments

## Data Availability

All the data included in the article are available to the public.

## SUPPLEMENTAL MATERIAL

OPEN PEER REVIEW reviewer-comments.pdf An accounting of the reviewer comments and feedback.

ASM does not own the copyrights to Supplemental Material that may be linked to, or accessed through, an article. The authors have granted ASM a non-exclusive, world-wide license to publish the Supplemental Material files. Please contact the corresponding author directly for reuse.
