# Supplementary material for: An evaluation of Innowave MTB/RIF/INH assay versus Xpert MTB/RIF for rapid identification of Mycobacterium tuberculosis and drug resistance in bone and joint specimens from people with suspected osteoarticular tuberculosis
Source: Microbiol Spectr. 2026 May 18;14(7):e00267-26. doi: 10.1128/spectrum.00267-26 (PMC13340047; doi:10.1128/spectrum.00267-26)
Supplement: Reviewer comments [file reviewer-comments.pdf]

AMERICAN SOCIETY FOR MICROBIOLOGY | Microbiology Spectrum

An evaluation of Innowave MTB/RIF/INH assay versus Xpert MTB/RIF for rapid identification of Mycobacterium tuberculosis and drug resistance in bone and joint specimens from people with suspected osteoarticular tuberculosis

Suting Chen, Qiang Liu, Fen Wang, Haoran Wang, Guanglu Jiang, Adong Shen, and Hairong Huang

Corresponding Author(s): Suting Chen, Beijing Chest Hospital Affiliated to Capital Medical University

**Review Timeline:**		
	Submission Date:	January 23, 2026
	Editorial Decision:	February 27, 2026
	Revision Received:	March 18, 2026
	Editorial Decision:	March 26, 2026
	Revision Received:	March 29, 2026
	Accepted:	April 3, 2026

Editor: Sophia Georghiou

Reviewer(s): Disclosure of reviewer identity is with reference to reviewer comments included in decision letter(s). The following individuals involved in review of your submission have agreed to reveal their identity: Kyung-Wook Jo (Reviewer #1); Kevin A Nash (Reviewer #2)

Transaction Report:

(Note: With the exception of the correction of typographical or spelling errors that could be a source of ambiguity, letters and reports are not edited. The original formatting of letters and referee reports may not be reflected in this compilation.)

DOI: https://doi.org/10.1128/spectrum.00267-26

Re: Spectrum00267-26 (**Head-to-head evaluation of Innowave MTB/RIF/INH assay versus Xpert MTB/RIF and culture for rapid diagnosis of osteoarticular tuberculosis and drug resistance**)

Dear Prof. Suting Chen:

Thank you for the privilege of reviewing your work. Below you will find instructions from the Spectrum editorial office and the reviewer comments.

Please return the manuscript within 60 days; if you cannot complete the modification within this time period, please contact me. If you do not wish to modify the manuscript and prefer to submit it to another journal, notify me immediately so that the manuscript may be formally withdrawn from consideration by Spectrum.

Revision Guidelines
To submit your modified manuscript, log into the submission site at https://spectrum.msubmit.net/cgi-bin/main.plex. Go to Author Tasks and click the appropriate manuscript title to begin. The information you entered when you first submitted the paper will be displayed; update this as necessary. Note the following requirements:

• Upload point-by-point responses to the issues raised by the reviewers in a file named "Response to Reviewers," NOT in your cover letter.
• Upload a compare copy of the manuscript (without figures) as a "Marked-Up Manuscript" file.
• Upload a clean .DOC/.DOCX version of the revised manuscript and remove the previous version.
• Each figure must be uploaded as a separate, editable, high-resolution file (TIFF or EPS preferred), and any multipanel figures must be assembled into one file.
• Any supplemental material intended for posting by ASM should be uploaded with their legends separate from the main manuscript. You can combine all supplemental material into one file (preferred) or split it into a maximum of 10 files with all associated legends included.

For complete guidelines on revision requirements, see our Submission and Review Process webpage. Submission of a paper that does not conform to guidelines may delay acceptance of your manuscript.

Data availability: ASM policy requires that data be available to the public upon online posting of the article, so please verify all links to sequence records, if present, and make sure that each number retrieves the full record of the data. If a new accession number is not linked or a link is broken, provide Spectrum production staff with the correct URL for the record. If the accession numbers for new data are not publicly accessible before the expected online posting of the article, publication may be delayed; please contact production staff (Spectrum@asmusa.org) immediately with the expected release date.

Publication Fees: For information on publication fees and which article types are subject to charges, visit our website. If your manuscript is accepted for publication and any fees apply, you will be contacted separately about payment during the production process; please follow the instructions in that e-mail. Arrangements for payment must be made before your article is published.

ASM Membership: Corresponding authors may join or renew ASM membership to obtain discounts on publication fees. Need to upgrade your membership level? Please contact Customer Service at Service@asmusa.org.

The ASM Journals program strives for constant improvement in our submission and publication process. Please tell us how we can improve your experience by taking this quick Author Survey.

Thank you for submitting your paper to Spectrum.

Sincerely,
Sophia Georghiou
Editor
Microbiology Spectrum

Reviewer #1 (Comments for the Author):

This is a well-organized and clearly presented prospective diagnostic study addressing a clinically important issue in osteoarticular tuberculosis. The manuscript is generally well structured, and the methodology and results are described in a coherent and accessible manner. I commend the authors for conducting a head-to-head comparison study in this challenging clinical setting. I have provided several additional comments below that I believe will further strengthen the methodological rigor and clarity of the manuscript.

1. For the central premise of this study to be fully justified, it would be important to establish that the prevalence of isoniazid (INH) resistance is relatively high in extrapulmonary tuberculosis (EPTB), particularly in osteoarticular TB. The clinical significance of detecting INH resistance using InnowaveDX depends largely on this assumption.
Are there prior studies demonstrating a higher rate of INH resistance in EPTB, especially in osteoarticular TB? If such evidence exists, incorporating it into the Introduction would substantially strengthen the rationale and highlight the clinical relevance of the present study.

2. The composite reference standard (CRS) used in this study includes smear microscopy, culture, and Xpert MTB/RIF results. Since Xpert is one of the index tests being evaluated, its inclusion in the CRS may introduce incorporation bias.
1) In diagnostic accuracy studies, the reference standard should ideally be independent of the index test. Including Xpert as part of the CRS may artificially influence its estimated sensitivity and specificity and compromise the independence of the head-to-head comparison between Xpert and InnowaveDX. The authors should clarify how this potential incorporation bias was addressed. Alternatively, analyses using a reference standard independent of Xpert (e.g., culture-confirmed cases or a CRS excluding Xpert results) would strengthen the validity of the comparison.
2) In addition, please clarify whether there were any cases in which smear microscopy was positive but both culture and Xpert MTB/RIF were negative. Such cases may represent nontuberculous mycobacterial infection rather than Mycobacterium tuberculosis. If such cases were present, it would be important to describe how they were handled in the analysis. Inclusion of smear-positive, culture-negative, and Xpert-negative cases without further clarification may introduce misclassification bias.

3. The reported specificity of InnowaveDX was 93.75% (45/48), indicating three false-positive results. Given the relatively low pretest probability of osteoarticular TB in such settings and the significant clinical implications of a positive molecular test, these false-positive cases warrant further clarification.
Please provide detailed information regarding these three cases, including their final clinical diagnoses, histopathological findings, follow-up data, and whether repeat testing was performed. It would also be helpful to discuss potential explanations for false-positive results.
In addition, the manuscript states that an open-format version of the assay was used rather than a fully closed cartridge-based system. Open-format molecular assays may be more susceptible to contamination or non-specific amplification. The authors should discuss whether laboratory contamination, IS6110 cross-reactivity, or detection of non-viable bacilli could have contributed to the observed false-positive results.

4. The authors included patients with concomitant pulmonary tuberculosis in the study population. However, when pulmonary TB and osteoarticular TB coexist, this condition is generally considered disseminated TB rather than isolated osteoarticular disease. Given the potentially different bacillary burden, immunologic background, and diagnostic yield between disseminated TB and isolated osteoarticular TB, analyzing these patients together may introduce clinical and microbiological heterogeneity. I suggest that patients with concomitant pulmonary TB be excluded from the primary analysis to ensure a more homogeneous study population focusing specifically on isolated osteoarticular TB.

5. In addition, it would be helpful for the authors to provide information regarding the analytical sensitivity (limit of detection) of the InnowaveDX assay, particularly the minimum detectable bacterial load (e.g., CFU/mL or genomic copies per reaction).

6. The core design of this study is (1) to compare InnowaveDX and Xpert in terms of diagnostic sensitivity and specificity for MTB detection, and (2) to assess the additional value of InnowaveDX in simultaneously detecting isoniazid (INH) resistance.
If similar head-to-head evaluations have already been conducted in pulmonary TB-particularly regarding the incremental benefit of adding INH resistance detection beyond Xpert-please briefly summarize the existing evidence in the Introduction and clearly state what remains unknown and how the present study fills that gap.

7. The rationale for performing the MeltPro TB test is not clearly described in the manuscript. It would be helpful for the authors to clarify why MeltPro was included in the study design.
Specifically, was MeltPro used as a reference method for drug resistance detection, as a comparator to InnowaveDX, or as part of routine clinical practice?

8. In Figure 1, the patient flow diagram would benefit from a clearer description of the inclusion and exclusion criteria applied at each step. It is not entirely clear how patients were screened, how many were excluded, and for what specific reasons. Please provide more detailed information on the enrollment process, including explicit inclusion and exclusion criteria and the reasons for exclusion.

9. The reported sensitivity of InnowaveDX for detecting rifampicin resistance (80%, 4/5) and isoniazid resistance (75%, 6/8) is based on a very small number of resistant isolates. With such limited sample sizes, the corresponding confidence intervals are likely to be wide, and the precision of these estimates is inherently low.
However, in the Discussion, the results are described using relatively strong language (e.g., "aligned perfectly" for rifampicin resistance and "demonstrated good predictive value" for isoniazid resistance). Given the small number of resistant cases and the moderate sensitivity observed, these statements appear somewhat overstated.
I recommend that the authors tone down the conclusions regarding drug resistance detection and explicitly acknowledge the limited statistical power and uncertainty associated with these resistance analyses.

Reviewer #2 (Comments for the Author):

This manuscript describes a small study of the utility of a novel molecular test, Innowave MTB/RIF/INH, compared with Xpert MTB/RIF and routine microbiological tests, to aid the diagnosis of osteoarticular TB.

Whilst members of this group have recently published studies elsewhere (Ann Clin Microbiol Antimicrob 24:52 and Diagn Microbiol Infect Dis 113:117059) on the Innowave technology, the novelty of this study is that it focuses on a problematic area of TB diagnosis, that is paucibacillary extrapulmonary TB represented by osteoarticular (musculoskeletal) disease.

For a small prospective proof of principle study, the work was well designed, and the results indicated that the Innowave test is marginally more sensitive than Xpert MTB/RIF at detecting MTB; however, this is not a particularly novel finding as there are many publications showing NAT technologies that perform better than the Xpert MTB/RIF at detecting MTB in clinical material. The key takeaways from this study are: (1) both the Xpert and Innowave tests perform remarkably better than culture at detecting MTB in bone and joint specimens in cases of osteoarticular TB; and, (2) Innowave is able to detect most rifampin resistance and some isoniazid resistance concurrently with MTB (note that the Xpert system does not assess isoniazid resistance).

I do have some concerns with the broader significance of the results as the version of the Innowave technology used in the study was the more labor intensive, more prone to contamination, open-format version. As the authors point out, this may explain the 96% specificity. Also, the comparison between the sensitivity of the Innowave and Xpert technologies was not a "level playing field" and biased in favor of the former.

The study would have more impact if sufficient data from the automated, closed-tube configuration of the Innowave MTB/RIF/INH technology used with the bone and joint specimens to demonstrate that is equivalent to the open-format version in the diagnosis of osteoarticular TB.

Other, minor, comments are listed below.
1. Title - The title is misleading - since both Innowave and Xpert detected MTB in all the culture positive specimens, the experimental comparison is not Innowave versus Xpert + culture. I suggest "An evaluation of Innowave MTB/RIF/INH assay versus Xpert MTB/RIF for rapid identification of Mycobacterium tuberculosis and drug resistance in bone and joint specimens from people with osteoarticular tuberculosis" or something similar.
2. Line 25 - change "pDST" to "DST" - this is the only place that pDST is used.
3. Lines 29, 92, & 148 - omit "consecutively"; or explain why it is important.
4. Lines 31 to 32 - omit the sentence beginning "Confirmed-TB detection rose..."; the important finding is summarized in the preceding sentence.
5. Line 97 - change "simultaneously" to "concurrently".
6. Line 125 - how was the DNA extracted? Following manufacturers instructions? Or as described elsewhere (with citation)?.
7. Line 127 - some explanation of what the "frozen product" was is needed - I assume that this is the Innowave PCR reagent cocktail, if so state that.
8. Line 134 - change "deviations" to "differences".
9. Line 136 - I prefer to use the term breakpoint concentrations rather than critical concentrations when referring to resistance thresholds.
10. Line 141 - some explanation of "other defined categories" is needed.
11. Line 148 - "Figure 1" should be "Table 1"
12. Line 153 - Since this is not a study of the incidence or prevalence of osteoarticular TB, this general statement is misleading. What the authors meant is clinical samples were obtained from a wider age range of individuals with confirmed/probable/possible osteoarticular TB than other musculoskeletal diagnoses. This is a sampling issue not a prevalence issue. Also, the manifestation of some diseases is significantly different in children than adults, I suggest omitting the pediatric cases or analyzing them separately.
13. Line 159 - A minor semantic issue, but MTB is not detected by smear - AFB are.
14. Line 171 - Although the Venn diagrams are pretty, I think the text is sufficient and so recommend omitting them.
15. Lines 175 & 185 - change "sensitive" to "sensitivity".
16. Line 194 - change "positive" to "concordant"; using the word positive in this context is ambiguous.
17. Line 196 - omit " predict" and change "consistency" to "concordance".
18. Line 198 - Change this sentence to "However, the concordance of the Meltpro INH assay was only 62.96% (17 out of 27)".
19. Line 204 - omit "an ancient"; this is not germane to this study.
20. Lines 213 to 214 - I would avoid a detailed re-stating of results in the Discussion section; it would be better to just say InnowaveDX achieved a significantly greater sensitivity than Xpert...
21. Line 229 - I recommend using paucibacillary rather than "low-bacterial-load".
22. Line 251 - missing 's' from symptoms.
23. Lines 257 to 266 - since this is really a proof of principle study most of the limitations are acceptable. As stated earlier, the

Innowave assay used in this study was not truly comparable to the Xpert MTB/RIF assay and the study would be much stronger if data using the Innowave automated, closed-tube configuration were included.

Tables and Figures.
All the tables are acceptable, but I recommend omitting the figures as the text is sufficient.

Reviewer #1 (Comments for the Author):

This is a well-organized and clearly presented prospective diagnostic study addressing a clinically important issue in osteoarticular tuberculosis. The manuscript is generally well structured, and the methodology and results are described in a coherent and accessible manner. I commend the authors for conducting a head-to-head comparison study in this challenging clinical setting. I have provided several additional comments below that I believe will further strengthen the methodological rigor and clarity of the manuscript.

Response: We would like to express our gratitude to you for your positive feedback on the research design, significance, and overall structure of the article. We have incorporated your valuable suggestions to revise the manuscript, striving to enhance its methodological rigor and improve coherence. The specific modifications are detailed below.

1. For the central premise of this study to be fully justified, it would be important to establish that the prevalence of isoniazid (INH) resistance is relatively high in extrapulmonary tuberculosis (EPTB), particularly in osteoarticular TB. The clinical significance of detecting INH resistance using InnowaveDX depends largely on this assumption.

Are there prior studies demonstrating a higher rate of INH resistance in EPTB, especially in osteoarticular TB? If such evidence exists, incorporating it into the Introduction would substantially strengthen the rationale and highlight the clinical relevance of the present study.

Response: The culture positivity rate for extrapulmonary tuberculosis (EPTB) is significantly lower than that for pulmonary tuberculosis (PTB). However, based on phenotypic drug sensitivity results, the rate of isoniazid resistance in EPTB appears to be comparable to that in PTB, although there is currently no direct research data to confirm this. The primary focus of our study is the substantial value of molecular diagnostic techniques in diagnosing EPTB cases. The InnowaveDX technology evaluated in this study can simultaneously detect Mycobacterium tuberculosis (Mtb) and assess its resistance to rifampicin and isoniazid. This capability can provide better guidance for clinical adjustments to anti-tuberculosis treatment plans.

2. The composite reference standard (CRS) used in this study includes smear microscopy, culture, and Xpert MTB/RIF results. Since Xpert is one of the index tests being evaluated, its inclusion in the CRS may introduce incorporation bias.

1) In diagnostic accuracy studies, the reference standard should ideally be independent of the index test. Including Xpert as part of the CRS may artificially influence its estimated sensitivity and specificity and compromise the independence of the head-to-head comparison between Xpert and InnowaveDX. The authors should clarify how this potential incorporation bias was addressed. Alternatively, analyses using a reference standard independent of Xpert (e.g., culture-confirmed cases or a CRS excluding Xpert results) would strengthen the validity of the comparison.

Response: We appreciate the reviewer's suggestion. We acknowledge that including the Xpert results in the CRS evaluation may introduce certain biases. Consequently, Table 3 presents the detection sensitivities of the two molecular testing methods in cases

confirmed by culture, with both methods demonstrating a sensitivity of 100%.

2) In addition, please clarify whether there were any cases in which smear microscopy was positive but both culture and Xpert MTB/RIF were negative. Such cases may represent nontuberculous mycobacterial infection rather than Mycobacterium tuberculosis. If such cases were present, it would be important to describe how they were handled in the analysis. Inclusion of smear-positive, culture-negative, and Xpert-negative cases without further clarification may introduce misclassification bias.

Response: Thank you for your suggestion. In this study, three cases tested positive on the smear test but returned negative results on both culture and Xpert. Based on the subsequent treatment outcomes of these patients, it was speculated that they might be infected with non-tuberculous mycobacteria (NTM). Therefore, we classified these three patients as non-tuberculosis cases for analysis, in order to rule out the possibility that the evaluated method might incorrectly identify NTM as Mycobacterium tuberculosis (Mtb). In the results section, we also provided a description of the test results for these three suspected NTM cases. Please see Lines 178-182.

3. The reported specificity of InnowaveDX was 93.75% (45/48), indicating three false-positive results. Given the relatively low pretest probability of osteoarticular TB in such settings and the significant clinical implications of a positive molecular test, these false-positive cases warrant further clarification.

Please provide detailed information regarding these three cases, including their final clinical diagnoses, histopathological findings, follow-up data, and whether repeat testing was performed. It would also be helpful to discuss potential explanations for false-positive

results.

In addition, the manuscript states that an open-format version of the assay was used rather than a fully closed cartridge-based system. Open-format molecular assays may be more susceptible to contamination or non-specific amplification. The authors should discuss whether laboratory contamination, IS6110 cross-reactivity, or detection of non-viable bacilli could have contributed to the observed false-positive results.

Response: Thank you for your suggestion. The final diagnoses for the three cases that were incorrectly identified as positive by InnowaveDX are as follows: one case involved suppurative spondylitis caused by Propionibacterium acnes infection, another was a case of suppurative arthritis caused by Klebsiella pneumoniae infection, and the third case was a secondary malignant tumor of the sacrum. We have added to the discussion section that open-format molecular assays may have limitations, such as laboratory contamination, IS6110 cross-reactivity, or false detection of dead bacteria, all of which can contribute to false positive results. Please see Lines 165-170 and Lines 236-240.

4. The authors included patients with concomitant pulmonary tuberculosis in the study population. However, when pulmonary TB and osteoarticular TB coexist, this condition is generally considered disseminated TB rather than isolated osteoarticular disease.

Given the potentially different bacillary burden, immunologic background, and diagnostic yield between disseminated TB and isolated osteoarticular TB, analyzing these patients together may introduce clinical and microbiological heterogeneity. I suggest that patients with concomitant pulmonary TB be excluded from the primary analysis to ensure a more homogeneous study population focusing specifically on isolated osteoarticular TB.

Response: Thank you. Theoretically, combining patients with pulmonary tuberculosis could lead to a larger bacterial load, but there is currently no definitive data to support this claim. In clinical practice, some patients do have co-infections. In this study, we did not specifically exclude patients with both tuberculosis and osteoarticular infections. This decision was primarily made because our comparisons of different diagnostic techniques were based on local lesion samples, such as tissue or pus.

5. In addition, it would be helpful for the authors to provide information regarding the analytical sensitivity (limit of detection) of the InnowaveDX assay, particularly the minimum detectable bacterial load (e.g., CFU/mL or genomic copies per reaction).

Response: Thank you very much. The detection limits for the InnowaveDX assay, as outlined in the Methods section, are as follows: The minimum detection limit for Mycobacterium tuberculosis (Mtb) is 10 bacteria per milliliter (bacteria/mL). For detecting rifampicin resistance, the minimum limit is 1000 colony-forming units per milliliter (CFU/mL), and the same minimum limit of 1000 CFU/mL applies for isoniazid resistance. Please see Lines 127-130.

6. The core design of this study is (1) to compare InnowaveDX and Xpert in terms of diagnostic sensitivity and specificity for MTB detection, and (2) to assess the additional value of InnowaveDX in simultaneously detecting isoniazid (INH) resistance.

If similar head-to-head evaluations have already been conducted in pulmonary TB-particularly regarding the incremental benefit of adding INH resistance detection beyond Xpert-please briefly summarize the existing evidence in the Introduction and clearly state what remains unknown and how the present study fills that gap.

Response: Thank you for your suggestion. In the Introduction section, we have revised the core design of the study and summarized the evaluation progress of Innowave DX diagnosis. Please see Lines 82-87.

7. The rationale for performing the MeltPro TB test is not clearly described in the manuscript. It would be helpful for the authors to clarify why MeltPro was included in the study design.

Specifically, was MeltPro used as a reference method for drug resistance detection, as a comparator to InnowaveDX, or as part of routine clinical practice?

Response: Thanks for the valuable suggestion. We have included the reasons for conducting the MeltPro TB test in the manuscript. In this study, the MeltPro test is capable of detecting Mycobacterium tuberculosis (Mtb) as well as isoniazid resistance. Therefore, it serves as a comparative method to assess the efficacy of the InnowaveDX in detecting isoniazid resistance. Please see Lines 197-199.

8. In Figure 1, the patient flow diagram would benefit from a clearer description of the inclusion and exclusion criteria applied at each step. It is not entirely clear how patients were screened, how many were excluded, and for what specific reasons. Please provide more detailed information on the enrollment process, including explicit inclusion and exclusion criteria and the reasons for exclusion.

Response: Thanks a lot. In revised Figure 1, we have added the inclusion and exclusion criteria, and also provided the number and reasons for the excluded patients.

9. The reported sensitivity of InnowaveDX for detecting rifampicin resistance (80%, 4/5) and isoniazid resistance (75%, 6/8) is based on a very small number of resistant isolates.

With such limited sample sizes, the corresponding confidence intervals are likely to be wide, and the precision of these estimates is inherently low.

However, in the Discussion, the results are described using relatively strong language (e.g., "aligned perfectly" for rifampicin resistance and "demonstrated good predictive value" for isoniazid resistance). Given the small number of resistant cases and the moderate sensitivity observed, these statements appear somewhat overstated.

I recommend that the authors tone down the conclusions regarding drug resistance detection and explicitly acknowledge the limited statistical power and uncertainty associated with these resistance analyses.

Response: Thank you for your suggestion. We tone down the conclusions regarding drug resistance detection since the number of samples available for analyzing drug resistance sensitivity and specificity is relatively limited. And also, we explicitly acknowledge the limited statistical power and uncertainty associated with these resistance analyses in the discussion section. Please see Lines254, 261-267.

Reviewer #2 (Comments for the Author):

This manuscript describes a small study of the utility of a novel molecular test, Innowave MTB/RIF/INH, compared with Xpert MTB/RIF and routine microbiological tests, to aid the diagnosis of osteoarticular TB.

Whilst members of this group have recently published studies elsewhere (Ann Clin

Microbiol Antimicrob 24:52 and Diagn Microbiol Infect Dis 113:117059) on the Innowave technology, the novelty of this study is that it focuses on a problematic area of TB diagnosis, that is paucibacillary extrapulmonary TB represented by osteoarticular (musculoskeletal) disease.

For a small prospective proof of principle study, the work was well designed, and the results indicated that the Innowave test is marginally more sensitive than Xpert MTB/RIF at detecting MTB; however, this is not a particularly novel finding as there are many publications showing NAT technologies that perform better than the Xpert MTB/RIF at detecting MTB in clinical material. The key takeaways from this study are: (1) both the Xpert and Innowave tests perform remarkably better than culture at detecting MTB in bone and joint specimens in cases of osteoarticular TB; and, (2) Innowave is able to detect most rifampin resistance and some isoniazid resistance concurrently with MTB (note that the Xpert system does not assess isoniazid resistance).

I do have some concerns with the broader significance of the results as the version of the Innowave technology used in the study was the more labor intensive, more prone to contamination, open-format version. As the authors point out, this may explain the 96% specificity. Also, the comparison between the sensitivity of the Innowave and Xpert technologies was not a "level playing field" and biased in favor of the former.

The study would have more impact if sufficient data from the automated, closed-tube configuration of the Innowave MTB/RIF/INH technology used with the bone and joint

specimens to demonstrate that is equivalent to the open-format version in the diagnosis of osteoarticular TB.

Response: Thank you for your valuable suggestion. In the Introduction section, we have included the assessment results of our team regarding InnowaveDX in the pulmonary tuberculosis population and provided the relevant reference, highlighting the novelty of this study. I also agree with the reviewer's opinion that we cannot simply assume that the sensitivity of InnowaveDX is greater than that of Xpert. The current version of InnowaveDX utilizes an open detection system, which, while increasing detection sensitivity, also makes it more susceptible to contamination, thereby potentially decreasing specificity. Therefore, any parallel comparison of the two technologies is likely to be influenced by certain biases. In the discussion section, we also emphasized the need for further evaluation of the closed-mode detection of this technology in the future. This would allow for a more objective analysis and comparison of sensitivity and specificity with Xpert technology. Please see Lines236-240.

Other, minor, comments are listed below.

1. Title - The title is misleading - since both Innowave and Xpert detected MTB in all the culture positive specimens, the experimental comparison is not Innowave versus Xpert + culture. I suggest "An evaluation of Innowave MTB/RIF/INH assay versus Xpert MTB/RIF for rapid identification of Mycobacterium tuberculosis and drug resistance in bone and joint specimens from people with osteoarticular tuberculosis" or something similar.

Response: Thank you for your good suggestion. We have updated the title of the

manuscript according to the suggestions of the reviewers.

2. Line 25 - change "pDST" to "DST" - this is the only place that pDST is used.

Response: Thanks. We have adopted the suggestions made by the reviewers.

3. Lines 29, 92, & 148 - omit "consecutively"; or explain why it is important.

Response: It has been revised as per the suggestions.

4. Lines 31 to 32 - omit the sentence beginning "Confirmed-TB detection rose..."; the important finding is summarized in the preceding sentence.

Response: It has been revised as per the suggestions.

5. Line 97 - change "simultaneously" to "concurrently".

Response: It has been revised as per the suggestions.

6. Line 125 - how was the DNA extracted? Following manufacturers instructions? Or as described elsewhere (with citation)?

Response: Thank you. We have enhanced the description of the DNA extraction method.

7. Line 127 - some explanation of what the "frozen product" was is needed - I assume that this is the Innowave PCR reagent cocktail, if so state that.

Response: Yes, this is the freeze-dried Innowave PCR reagent mix. We have revised this sentence.

8. Line 134 - change "deviations" to "differences".

Response: It has been revised as per the suggestion.

9. Line 136 - I prefer to use the term breakpoint concentrations rather than critical concentrations when referring to resistance thresholds.

Response: It has been revised as per the suggestion.

10. Line 141 - some explanation of "other defined categories" is needed.

Response: Thank you. We have added supplementary descriptions for "other defined categories".

11. Line 148 - "Figure 1" should be "Table 1"

Response: Sorry for this mistake.

12. Line 153 - Since this is not a study of the incidence or prevalence of osteoarticular TB, this general statement is misleading. What the authors meant is clinical samples were obtained from a wider age range of individuals with confirmed/probable/possible osteoarticular TB than other musculoskeletal diagnoses. This is a sampling issue not a prevalence issue. Also, the manifestation of some diseases is significantly different in children than adults, I suggest omitting the pediatric cases or analyzing them separately.

Response: Thank you for your suggestion. This study does not focus on the incidence or prevalence rates of bone tuberculosis. Therefore, simply comparing the ages of the bone tuberculosis group with the non-bone tuberculosis group is neither accurate nor meaningful. As a result, we have decided to remove this description from the results.

13. Line 159 - A minor semantic issue, but MTB is not detected by smear - AFB are.

Response: Thank you for your suggestion. We made the necessary revisions according to it to make the expression more accurate.

14. Line 171 - Although the Venn diagrams are pretty, I think the text is sufficient and so recommend omitting them.

Response: Thank you. We have removed the Venn diagram.

15. Lines 175 & 185 - change "sensitive" to "sensitivity".

Response: Thank you. The correction has been made.

16. Line 194 - change "positive" to "concordant"; using the word positive in this context is ambiguous.

Response: Thank you. The correction has been made.

17. Line 196 - omit " predict" and change "consistency" to "concordance".

Response: Thank you. The correction has been made.

18. Line 198 - Change this sentence to "However, the concordance of the Meltpro INH assay was only 62.96% (17 out of 27)".

Response: Thank you. The correction has been made.

19. Line 204 - omit "an ancient"; this is not germane to this study.

Response: Thank you. It has been deleted.

20. Lines 213 to 214 - I would avoid a detailed re-stating of results in the Discussion section; it would be better to just say InnowaveDX achieved a significantly greater sensitivity than Xpert...

Response: Thank you. The correction has been made.

21. Line 229 - I recommend using paucibacillary rather than "low-bacterial-load".

Response: Thank you. The correction has been made.

22. Line 251 - missing 's' from symptoms.

Response: Thank you. It has been updated.

23. Lines 257 to 266 - since this is really a proof of principle study most of the limitations are acceptable. As stated earlier, the Innowave assay used in this study was not truly comparable to the Xpert MTB/RIF assay and the study would be much stronger if data

using the Innowave automated, closed-tube configuration were included.

Response: Thank you for your constructive suggestions. We will evaluate the InnowaveDX automated closed-tube configuration and compare it with the Xpert MTB/RIF in our upcoming study.

Re: Spectrum00267-26R1 (**An evaluation of Innowave MTB/RIF/INH assay versus Xpert MTB/RIF for rapid identification of Mycobacterium tuberculosis and drug resistance in bone and joint specimens from people with suspected osteoarticular tuberculosis**)

Dear Prof. Suting Chen:

Thank you for the privilege of reviewing your work. Below you will find my comments, instructions from the Spectrum editorial office, and the reviewer comments. Overall, the Reviewers identified only a few remaining, minor issues to be addressed.

Please return the manuscript within 60 days; if you cannot complete the modification within this time period, please contact me. If you do not wish to modify the manuscript and prefer to submit it to another journal, notify me immediately so that the manuscript may be formally withdrawn from consideration by Spectrum.

Revision Guidelines
To submit your modified manuscript, log into the submission site at https://spectrum.msubmit.net/cgi-bin/main.plex. Go to Author Tasks and click the appropriate manuscript title to begin. The information you entered when you first submitted the paper will be displayed; update this as necessary. Note the following requirements:

• Upload point-by-point responses to the issues raised by the reviewers in a file named "Response to Reviewers," NOT in your cover letter.
• Upload a compare copy of the manuscript (without figures) as a "Marked-Up Manuscript" file.
• Upload a clean .DOC/.DOCX version of the revised manuscript and remove the previous version.
• Each figure must be uploaded as a separate, editable, high-resolution file (TIFF or EPS preferred), and any multipanel figures must be assembled into one file.
• Any supplemental material intended for posting by ASM should be uploaded with their legends separate from the main manuscript. You can combine all supplemental material into one file (preferred) or split it into a maximum of 10 files with all associated legends included.

For complete guidelines on revision requirements, see our Submission and Review Process webpage. Submission of a paper that does not conform to guidelines may delay acceptance of your manuscript.

Data availability: ASM policy requires that data be available to the public upon online posting of the article, so please verify all links to sequence records, if present, and make sure that each number retrieves the full record of the data. If a new accession number is not linked or a link is broken, provide Spectrum production staff with the correct URL for the record. If the accession numbers for new data are not publicly accessible before the expected online posting of the article, publication may be delayed; please contact production staff (Spectrum@asmusa.org) immediately with the expected release date.

Publication Fees: For information on publication fees and which article types are subject to charges, visit our website. If your manuscript is accepted for publication and any fees apply, you will be contacted separately about payment during the production process; please follow the instructions in that e-mail. Arrangements for payment must be made before your article is published.

ASM Membership: Corresponding authors may join or renew ASM membership to obtain discounts on publication fees. Need to upgrade your membership level? Please contact Customer Service at Service@asmusa.org.

The ASM Journals program strives for constant improvement in our submission and publication process. Please tell us how we can improve your experience by taking this quick Author Survey.

Thank you for submitting your paper to Spectrum.

Sincerely,
Sophia Georghiou
Editor
Microbiology Spectrum

Reviewer #1 (Comments for the Author):

The authors have provided an appropriate and satisfactory response to my comments. Thank you.

Reviewer #2 (Comments for the Author):

Although the manuscript is much improved and the authors satisfactorily addressed the comments of the previous reviews, there are several significant other issues (listed below) that should be addressed.

1. Line 126 - omit "freeze-dried"; the material was probably frozen (not freeze-dried) but stating this in the text is not necessary.
2. Line 151 - omit "finally".
3. Line 196 - change "INH susceptible detection" to "INH resistance detection"; the molecular assays to not detect susceptibility (unless of course the assays can detect all possible resistance-associated mutations).
4. Line 197 - change "isoniazid-sensitivity osteoarticular TB" to "isoniazid-sensitive osteoarticular TB".
5. Lines 236 to 240 - these sentences would be better placed in the paragraph on study limitations (lines 275 to 284). Also, "cross-reactivity with the IS6110 antigen, and misinterpretation of dead bacteria" are not likely to be issues of only the "open-tube" approach and thus I would omit mentioning them unless the authors believe that they are actual problems with their assay; plus it is not clear what the authors are inferring by "misinterpretation of dead bacteria"?

Reviewer #2 (Comments for the Author):

Although the manuscript is much improved and the authors satisfactorily addressed the comments of the previous reviews, there are several significant other issues (listed below) that should be addressed.

1. Line 126 - omit "freeze-dried"; the material was probably frozen (not freeze-dried) but stating this in the text is not necessary.

Response: Thank you. It has been revised.

2. Line 151 - omit "finally".

Response: Thank you. It has been deleted.

3. Line 196 - change "INH susceptible detection" to "INH resistance detection"; the molecular assays to not detect susceptibility (unless of course the assays can detect all possible resistance-associated mutations).

Response: Yes, it has been revised.

4. Line 197 - change "isoniazid-sensitivity osteoarticular TB" to "isoniazid-sensitive osteoarticular TB".

Response: Thank you. It has been revised.

5. Lines 236 to 240 - these sentences would be better placed in the paragraph on study limitations (lines 275 to 284). Also, "cross-reactivity with the IS6110 antigen, and misinterpretation of dead bacteria" are not likely to be issues of only the "open-tube" approach and thus I would omit mentioning them unless the authors believe that they are actual problems with their assay; plus it is not clear what the authors are inferring by "misinterpretation of dead bacteria"?

Response: Thank you for your suggestion. We have moved these sentences to the limitations section and revised them accordingly. Additionally, the concerns regarding "cross-reactivity with the IS6110 antigen and the misinterpretation of dead bacteria" are not exclusive to the "open-tube" approach; therefore, I have chosen to omit mentioning them.

Re: Spectrum00267-26R2 (**An evaluation of Innowave MTB/RIF/INH assay versus Xpert MTB/RIF for rapid identification of Mycobacterium tuberculosis and drug resistance in bone and joint specimens from people with suspected osteoarticular tuberculosis**)

Dear Prof. Suting Chen:

Your manuscript has been accepted, and I am forwarding it to the ASM production staff for publication. Your paper will first be checked to make sure all elements meet the technical requirements. ASM staff will contact you if anything needs to be revised before copyediting and production can begin. Otherwise, you will be notified when your proofs are ready to be viewed.

Data Availability: ASM policy requires that data be available to the public upon online posting of the article, so please verify all links to sequence records, if present, and make sure that each number retrieves the full record of the data. If a new accession number is not linked or a link is broken, provide production staff with the correct URL for the record. If the accession numbers for new data are not publicly accessible before the expected online posting of the article, publication may be delayed; please contact ASM production staff immediately with the expected release date.

Publication Fees: For information on publication fees and which article types have charges, please visit our website. We have partnered with Copyright Clearance Center (CCC) to collect author charges. If fees apply to your paper, you will receive a message from no-reply@copyright.com with further instructions. For questions related to paying charges through RightsLink, please contact CCC at ASM_Support@copyright.com or toll free at +1-877-622-5543. CCC makes every attempt to respond to all emails within 24 hours.

ASM Membership: Corresponding authors may join or renew ASM membership to obtain discounts on publication fees. Need to upgrade your membership level? Please contact Customer Service at Service@asmusa.org.

Embargo Policy: A press release may be issued as soon as the manuscript is posted on the Spectrum Latest Articles webpage. The corresponding author will receive an email with the subject line "ASM Journals Author Services Notification" when the article is available online.

The ASM Journals program strives for constant improvement in our submission and publication process. Please tell us how we can improve your experience by taking this quick Author Survey.

Thank you for submitting your paper to Spectrum.

Sincerely,
Sophia Georghiou
Editor
Microbiology Spectrum
